# Supplementary material for: Multigroup analysis of compositions of microbiomes with covariate adjustments and repeated measures
Source: Nat Methods. 2023 Dec 29;21(1):83–91. doi: 10.1038/s41592-023-02092-7 (PMC10776411; doi:10.1038/s41592-023-02092-7)
Supplement: Supplementary file 1 — Supplementary Methods and Table 1. [file 41592_2023_2092_MOESM1_ESM.pdf]

nature methods

Article https://doi.org/10.1038/s41592-023-02092-7

Multigroup analysis of compositions of microbiomes with covariate adjustments and repeated measures

In the format provided by the authors and unedited

Contents

1 Supplementary Methods **2**
1.1 Simulation Details 2
1.2 FDR Adjusted Power (FAP) 5
1.3 Computational Efficiency and Performance Benchmarking of Various DA Methods 5

2 Supplementary Tables **6**

1 Supplementary Methods

1.1 Simulation Details

The simulation studies conducted in this paper employed the Poisson lognormal (PLN) model to construct microbial absolute abundances, adhering to the methodology presented in the Linear Decomposition Model (LDM) [1]. The PLN model proposes that microbial abundance derives from a Poisson distribution, with the mean conforming to a multivariate log-normal distribution. This approach aligns the abundance vector with a Gaussian latent vector. The inclusion of a latent layer within the PLN model results in an elevated variance compared to a standard Poisson model, effectively capturing the over-dispersion traits inherent to microbiome data. Moreover, the covariance or correlation found among abundances mirrors the same covariance or correlation among the associated latent variables, with the underlying multivariate Gaussian distribution providing enhanced flexibility in the modeling of variance-covariance structures for microbial absolute abundances. For the construction of these simulations, we utilized a subset of the upper respiratory tract (URT) microbiome data, comprising 60 samples and 382 operational taxonomic units (OTUs), from an original dataset of 60 samples and 856 OTUs [2]. OTUs present in less than 5% of samples were excluded to refine the dataset. Rather than a manual specification of the mean vector and the variance-covariance matrix for absolute abundances, we employed estimates derived from the actual URT dataset, grounding our simulation parameters in real-world data. It is of critical importance to highlight that our methodology, ANCOM-BC2, was not derived from the PLN model; therefore, our simulation data does not intrinsically privilege our methodology in comparison to competing methods.

Informed by the observed shortcomings of ANCOM-BC from our practical application, we designed an exhaustive simulation study emphasizing scenarios that highlighted ANCOM-BC's limitations, aiming to evaluate the robustness of various DA methodologies. We incorporated two sources of bias into the synthetic data. We accounted for potential sequencing efficiency discrepancies by implementing a feature-specific bias for each taxon, which was randomly sampled from a uniform distribution ($C \sim U[0.1, 1]$). In parallel, a sample-specific bias was introduced to ensure the presence of rare taxa (exhibiting over 50% zeros across samples), providing a robustness test for the DA methods in terms of pseudo-count addition. This bias was closely correlated with the exposure of interest, thereby examining the methods' performance in the face of batch effects — a frequent concern in large-scale omics studies. Additional information about the simulation design for batch effects can be found in Extended Data Fig. 1. We integrated the influences of both the exposure and adjusting covariates into the log absolute abundance data for a certain proportion of taxa—referred to as DA or non-null taxa. The log fold-changes for these variables, based on the natural logarithm, were drawn from $[-2, -1, 1, 2]$, representing a fold-change varying from 0.14 to 7.4 on the original scale. We also investigated the DA methods' robustness in relation to the violation of an assumption pervasive in many DA methodologies: the assumption that most taxa are not differentially abundant. We examined five cases in which the underlying true proportion of DA taxa varied from 5% to 90% (p = 5%, 10%, 20%, 50%, and 90%). The selection of DA taxa was performed randomly. A broad span of sample sizes, or samples per group if the exposure is discrete, was examined: $n = 10, 20, 30, 50, 100$. For ANCOM-BC2, we employed a sensitivity analysis specific to the addition of pseudo-counts to zeros. Consequently, we introduced two variants of ANCOM-BC2: ANCOM-BC2 (No Filter) and ANCOM-BC2 (SS Filter). The former, ANCOM-BC2 (No Filter), is devised to identify significant taxa via the ANCOM-BC2 methodology, regardless of their performance in the sensitivity score filter. This includes taxa that display sensitivity to the inclusion of pseudo-counts.

Conversely, the ANCOM-BC2 (SS Filter) variant also utilizes the ANCOM-BC2 methodology for the identification of significant taxa, but it distinctly omits those that failed to meet the sensitivity score filter criteria. Here, a taxon would only be declared significant if it was significant without the addition of a pseudo-count (in line with the ANCOM-BC2 default setting) and if it remained significant across a range of pseudo-count additions. For the control of false discovery rates due to multiple testing, we favored the Holm-Bonferroni method [3] over the Benjamini-Hochberg (BH) procedure [4] for all DA methods. The Holm-Bonferroni method, independent of assumptions regarding the dependence structure among the underlying p-values, is recognized as more robust when dealing with inaccurate p-values [5]. In regard to data preprocessing, we have utilized a tailored protocol based on the DA methods employed. Specifically, for ANCOM-BC2, ANCOM-BC, LinDA, and LOCOM, a taxon filter was applied to exclude taxa displaying less than 10% prevalence across samples. Concurrently, samples with a library size (total counts across taxa) of less than or equal to 1000 counts were discarded from the study. In relation to the use of CORNCOB, a method specializing in differential relative abundance analysis, we also implemented an exclusionary criterion based on library size, specifically discarding any samples with counts of 1000 or less. It is important to note that the application of a taxon filter was not feasible within this method due to limitations inherent in its current implementation, which does not provide for this feature. In consideration of the computational efficiency of CORNCOB, the bootstrap function was disabled, and the Wald test statistic was employed. Beyond these specific modifications, the default parameters were retained in all DA methods unless otherwise stated, ensuring consistency in the analyses.

Continuous exposure. We generated a continuous exposure variable ($cont$) from a standard normal distribution ($cont \sim N(0,1)$) and introduced an adjusting binary covariate that equally divided the samples into two categories. The log fold-changes for the adjusting covariate, drawn from $[0,1]$, were applied to a randomly chosen subset of taxa. Sample-specific bias (S), reflective of sampling fraction differences, was set to strongly correlate with the continuous exposure ($S = \text{softmax}(cont)/10$) to simulate batch effects. The log-transformed sample-specific bias ($s = \log S$) and feature-specific bias ($c = \log C$) were applied to the log absolute abundances of samples and taxa, respectively. Each (n, p) combination was iterated 100 times, with a significance level of 0.05.

Binary exposure. In our simulation study concerning a binary exposure with a continuous adjusting covariate, we modified the settings used in the continuous exposure scenario. We evaluated a sequence of per-group sample sizes: $10, 20, 30, 50$, and 100, resulting in total sample sizes for the binary exposure of $20, 40, 60, 100$, and 200. To simulate batch effects, sample-specific bias (S) was strongly correlated with the binary exposure, set between $1e-4$ to $1e-3$ ($S \sim U[1e-4, 1e-3]$) for the first half of samples and $1e-3$ to $1e-2$ ($S \sim U[1e-3, 1e-2]$) for the second half. An adjusting continuous covariate was incorporated, with its log fold-changes drawn from $[0,1]$, and applied to a randomly selected taxa subset with non-zero log fold-changes.

Multiple pairwise comparisons (against a reference group). In our simulation study involving multiple pairwise comparisons against the reference group, we used the sample settings from the binary exposure scenario but with a three-level categorical variable as the exposure of interest and a continuous variable as the adjusting covariate. The sample sizes per group ranged from 10 to 100, resulting in total sample sizes for the categorical exposure between 30 and 300. To simulate batch effects, the sample-specific bias (S) was strongly correlated with the categorical

exposure, with S values ranging from $1e-4$ to $1e-3$ ($S \sim U[1e-4, 1e-3]$) for the first third of samples, $1e-3$ to $1e-2$ ($S \sim U[1e-3, 1e-2]$) for the middle third, and $1e-2$ to $1e-1$ ($S \sim U[1e-2, 1e-1]$) for the last third. The log fold-changes for DA (non-null) taxa in the second group compared to the reference group and in the third group compared to the reference group were randomly drawn from the range of [-2, -1, 1, 2]. The selection of DA taxa for the two comparisons was performed randomly and could differ between the comparisons.

Multiple pairwise comparisons. In our simulation of multiple pairwise comparisons, we utilized the sample settings from the multiple pairwise comparisons against the reference group scenario. However, we expanded the analysis to include comparisons between the second group and the third group. Specifically, the log fold-changes between the second group and the third group were calculated as the difference between the log fold-changes of "the second group compared to the reference group" and "the third group compared to the reference group".

Pattern analysis. In our simulation study for pattern analysis, we largely replicated the settings from the multiple pairwise comparisons study, with an exception made for the log fold-changes for the categorical exposure, tailored to benchmark a scenario featuring a monotonically increasing pattern. Here, the log fold-change between the second group and the reference group, denoted as δ, was randomly drawn from the set $[0.5, 1.0, 1.5, 2.0]$, and the log fold-change for the third group was fixed at $\delta + 1$ in relation to the reference group.

Correlated samples. In our simulation study, we contemplated two scenarios: one harboring solely a random intercept effect and another hosting both random intercept and random slope effects. Both random effects were characterized by a mean of zero, with standard deviations of 1 and 1.5 for the random intercept and random slope, respectively. If both random effects were present, they were associated with a correlation coefficient of 0.5. In these scenarios, the exposure variable contained three levels, thus defining three experimental groups. A continuous adjusting covariate was also incorporated. The remaining simulation settings were maintained as previously described in the preceding sections.

Normalization and transformation used for different DA methods We provide additional details regarding the preprocessing steps for each DA method discussed in our simulation studies. For ANCOM-BC2 and ANCOM-BC, no external normalization or transformation was applied to the input data. These methodologies internally estimate and correct biases, such as sample- and taxon-specific biases in ANCOM-BC2, and sample-specific bias in ANCOM-BC, prior to conducting statistical inferences. Therefore, these methods operate on internally "normalized" or "bias-corrected" absolute abundances. In the case of LinDA, it applies the centered Log-Ratio (CLR) transformation to the input data and incorporates an internal normalization procedure as part of its bias-correction process, akin to ANCOM-BC2 and ANCOM-BC. No further external transformation or normalization steps were performed for LinDA. LOCOM, on the other hand, accepts relative abundances (proportions) as input, which can be viewed as data already subjected to a "total-sum scaling" normalization. LOCOM infers changes in absolute abundance through a transformation similar to the Additive Log-Ratio (ALR) transformation. As such, LOCOM does not require any additional external transformation or normalization. CORNCOB, designed specifically for analyzing relative abundances, includes an internal "total-sum scaling" normalization procedure. No transformation is performed on the data for CORNCOB.

1.2 FDR Adjusted Power (FAP)

We introduced a novel concept, the "False Discovery Rate Adjusted Power (FAP)", defined as $ln\frac{\text{power}}{\text{FDR}}$. Consequently, a method with a lower FDR and higher power would achieve a higher FAP value, indicative of a good power/FDR trade-off. The FAP was computed using power and FDR values derived from the simulation studies conducted for both continuous and binary exposure scenarios discussed in Fig. 1 of the main paper. It should be noted that the FAP is an average of over 100 simulation runs at each setting. Note that methods with very low power but equally low FDR may also yield a high FAP. Hence, the selection of a method should not rely solely on a high FAP value, but the user should make FAP comparisons with a minimum power requirement, such as 0.6 or 0.8, etc.

1.3 Computational Efficiency and Performance Benchmarking of Various DA Methods

We evaluated the computational efficiency of the methods described in this paper using the "atlas1006" data [6]. Our focus was limited to comparing the "lean" and "obese" subjects since not all methods are tailored for multi-group hypothesis testing. Also, since not all methods considered in this paper are suitable for repeated measurements, we confined our comparisons to only baseline data. This resulted in 130 genera across 630 samples. The CPU time for implementing each method is summarized as follows: LOCOM and CORNCOB were computationally most intensive, requiring close to a minute each (62.3 seconds and 59.34 seconds, respectively), whereas ANCOM-BC2 (SS Filter) was faster than these two methods clocking in at 29.47 seconds. ANCOM-BC2 (No Filter) required only 8.58 seconds and ANCOM-BC took 6.02 seconds. Although LinDA was the fastest algorithm, taking only 0.05 seconds, it may not be preferable due to potentially high FDR as reported in simulation studies. Relative to the amount of time taken to conduct microbiome studies and generate data, the CPU time taken by the above DA methods is trivial. Hence when choosing a DA analysis method, characteristics such as FDR control and power should outweigh the CPU time taken by a method.

2 Supplementary Tables

Supplementary Table 1: Presence/Absence Test on the Soil Microbiome Data

**Genus**	**Arid**	**Margin**	**Hyperarid**
4W	Presence	Absence	Absence
A17	Presence	Presence	Absence
Acanthamoeba	Presence	Presence	Absence
Acidisoma	Presence	Absence	Absence
Acidisphaera	Presence	Absence	Absence
Acidocella	Absence	Presence	Absence
Acidovorax	Presence	Absence	Absence
Actinoallomurus	Presence	Absence	Absence
Actinocorallia	Presence	Absence	Absence
Actinomyces	Absence	Presence	Absence
Actinomycetospora	Presence	Presence	Absence
Actinophytocola	Presence	Presence	Absence
Actinoplanes	Presence	Presence	Absence
Actinopolymorpha	Presence	Presence	Absence
Aeromicrobium	Presence	Absence	Absence
Aetherobacter	Presence	Absence	Absence
Afipia	Presence	Absence	Absence
Agromyces	Presence	Absence	Absence
Alkaliflexus	Presence	Absence	Absence
Altererythrobacter	Presence	Absence	Absence
Aminobacter	Presence	Absence	Absence
Amorphomonas	Absence	Presence	Absence
Anaeromyxobacter	Presence	Presence	Absence
Ancylobacter	Presence	Absence	Absence
Aquicella	Presence	Presence	Absence
Aquincola	Presence	Absence	Absence
Ardenscatena	Absence	Presence	Absence
Arthrobacter	Presence	Presence	Absence
Arthrospira	Presence	Absence	Absence
Asteroleplasma	Presence	Absence	Absence
Azoarcus	Presence	Absence	Absence
Azohydromonas	Presence	Absence	Absence
Azospirillum	Absence	Presence	Absence
Bacillus	Presence	Absence	Absence
Balneimonas	Presence	Presence	Absence
Beijerinckia	Presence	Absence	Absence
Belnapia	Presence	Absence	Absence
Bifidobacterium	Presence	Absence	Absence
Blautia	Absence	Presence	Absence
Bosea	Presence	Absence	Absence
Buchnera	Absence	Presence	Absence

Continued on next page

Supplementary Table 1 – continued from previous page

Genus	Arid	Margin	Hyperarid
Byssovorax	Presence	Absence	Absence
Caldilinea	Presence	Absence	Absence
Caldimonas	Absence	Presence	Absence
Caloramator	Absence	Presence	Absence
Candidatus Amoebophilus	Presence	Absence	Absence
Candidatus Entotheonella	Presence	Presence	Absence
Candidatus Koribacter	Presence	Presence	Absence
Candidatus Protochlamydia	Presence	Absence	Absence
Candidatus Rhabdochlamydia	Presence	Absence	Absence
Candidatus Solibacter	Presence	Presence	Absence
Candidatus Xiphinematobacter	Presence	Absence	Absence
Carboxydothermus	Absence	Presence	Absence
Cardiobacterium	Presence	Absence	Absence
Catellatospora	Presence	Presence	Absence
Catenuloplanes	Presence	Absence	Absence
Caulobacter	Presence	Absence	Absence
Cellulomonas	Presence	Presence	Absence
Cesiribacter	Presence	Absence	Absence
Chelativorans	Presence	Presence	Absence
Chelatococcus	Absence	Presence	Absence
Chitinophaga	Presence	Presence	Absence
Chroococcidiopsis	Presence	Absence	Absence
Chthoniobacter	Presence	Presence	Absence
Chthonomonas	Presence	Absence	Absence
Clavisporangium	Presence	Absence	Absence
Cohnella	Presence	Presence	Absence
Collimonas	Presence	Absence	Absence
Conexibacter	Presence	Presence	Absence
Coxiella	Presence	Absence	Absence
Crocinitomix	Presence	Absence	Absence
Cryocola	Presence	Absence	Absence
Cupriavidus	Presence	Absence	Absence
Cytophaga	Presence	Absence	Absence
Dactylosporangium	Presence	Presence	Absence
Desmospora	Presence	Absence	Absence
Devosia	Presence	Presence	Absence
Dokdonella	Presence	Absence	Absence
Dongia	Presence	Absence	Absence
Dyadobacter	Presence	Absence	Absence
Dyella	Presence	Absence	Absence
Edaphobacter	Presence	Absence	Absence
Ellin506	Presence	Absence	Absence
Enhydrobacter	Presence	Absence	Absence
Enterobacter	Presence	Absence	Absence
Enterococcus	Absence	Presence	Absence

Continued on next page

Supplementary Table 1 – continued from previous page

Genus	Arid	Margin	Hyperarid
Erythrobacter	Presence	Absence	Absence
Estrella	Presence	Absence	Absence
FFCH4570	Presence	Presence	Absence
Filimonas	Presence	Absence	Absence
Fimbriimonas	Presence	Absence	Absence
Flavobacterium	Presence	Absence	Absence
Fluviicola	Presence	Presence	Absence
Fodinicola	Presence	Absence	Absence
Frigoribacterium	Absence	Presence	Absence
Fulvivirga	Presence	Absence	Absence
Gaiella	Presence	Absence	Absence
Georgenia	Absence	Presence	Absence
Glycomyces	Presence	Presence	Absence
Haliangium	Presence	Absence	Absence
Herminiimonas	Presence	Absence	Absence
heteroC45	Presence	Absence	Absence
Hymenobacter	Presence	Absence	Absence
Hyphomicrobium	Presence	Presence	Absence
Iamia	Presence	Presence	Absence
Inquilinus	Presence	Presence	Absence
Janthinobacterium	Presence	Presence	Absence
JG37-AG-70	Presence	Presence	Absence
Kaistia	Presence	Absence	Absence
Kaistibacter	Presence	Absence	Absence
Kibdelosporangium	Presence	Presence	Absence
Kitasatospora	Presence	Absence	Absence
Kribbella	Presence	Presence	Absence
Kutzneria	Presence	Absence	Absence
Kyrpidia	Presence	Absence	Absence
Labrys	Presence	Absence	Absence
Laceyella	Presence	Absence	Absence
Lachnoanaerobaculum	Presence	Absence	Absence
Larkinella	Presence	Absence	Absence
Legionella	Presence	Absence	Absence
Leminorella	Absence	Presence	Absence
Lentzea	Presence	Absence	Absence
Limnobacter	Presence	Absence	Absence
Listeria	Presence	Absence	Absence
Loktanella	Presence	Presence	Absence
Longispora	Presence	Absence	Absence
Luteibacter	Presence	Absence	Absence
Luteimonas	Presence	Absence	Absence
Luteolibacter	Presence	Absence	Absence
Lutibacterium	Absence	Presence	Absence
Lysobacter	Presence	Absence	Absence

Continued on next page

Supplementary Table 1 – continued from previous page

Genus	Arid	Margin	Hyperarid
Magnetospirillum	Presence	Presence	Absence
Maribacter	Presence	Absence	Absence
Marina	Presence	Absence	Absence
Marmoricola	Presence	Presence	Absence
Megamonas	Absence	Presence	Absence
Methylibium	Presence	Presence	Absence
Methylobacillus	Presence	Absence	Absence
Methylobacterium	Presence	Absence	Absence
Methylotenera	Presence	Absence	Absence
Microbispora	Presence	Absence	Absence
Microbispora	Presence	Presence	Absence
Micrococcus	Absence	Presence	Absence
Microlunatus	Presence	Absence	Absence
Micromonospora	Presence	Presence	Absence
Moryella	Absence	Presence	Absence
Mucilaginibacter	Presence	Absence	Absence
Mycobacterium	Presence	Presence	Absence
Mycoplana	Presence	Absence	Absence
Myxococcus	Presence	Absence	Absence
Nannocystis	Presence	Presence	Absence
Neochlamydia	Presence	Absence	Absence
Niabella	Presence	Absence	Absence
Niastella	Presence	Absence	Absence
Nitrobacter	Presence	Absence	Absence
Nitrosovibrio	Presence	Presence	Absence
Nocardia	Presence	Absence	Absence
Nocardioides	Presence	Presence	Absence
Nonomuraea	Presence	Presence	Absence
Nostocoida	Presence	Absence	Absence
Oceanobacillus	Presence	Absence	Absence
Olivibacter	Presence	Absence	Absence
Olsenella	Presence	Absence	Absence
Opitutus	Presence	Presence	Absence
OR-59	Presence	Absence	Absence
Oryzihumus	Presence	Absence	Absence
Oscillochloris	Presence	Absence	Absence
Oxalobacter	Presence	Absence	Absence
Parabacteroides	Presence	Absence	Absence
Parachlamydia	Presence	Absence	Absence
Paracraurococcus	Presence	Presence	Absence
Parasegitibacter	Presence	Absence	Absence
Pasteuria	Presence	Absence	Absence
Patulibacter	Presence	Absence	Absence
Paucibacter	Presence	Absence	Absence
Pedobacter	Presence	Presence	Absence

Continued on next page

Supplementary Table 1 – continued from previous page

Genus	Arid	Margin	Hyperarid
Pedomicrobium	Presence	Presence	Absence
Pedosphaera	Presence	Presence	Absence
Peredibacter	Presence	Absence	Absence
Phaeospirillum	Presence	Absence	Absence
Phaselicystis	Presence	Absence	Absence
Phenylobacterium	Presence	Presence	Absence
Phycicoccus	Presence	Absence	Absence
Phyllobacterium	Presence	Absence	Absence
Phytohabitans	Presence	Presence	Absence
Pilimelia	Presence	Absence	Absence
Pimelobacter	Presence	Presence	Absence
Pirellula	Presence	Presence	Absence
planctomycete	Presence	Absence	Absence
Pleomorphomonas	Presence	Presence	Absence
Plesiocystis	Presence	Absence	Absence
Polaromonas	Presence	Absence	Absence
Porphyrobacter	Presence	Absence	Absence
Procabacter	Presence	Absence	Absence
Promicromonospora	Presence	Absence	Absence
Prosthecobacter	Presence	Absence	Absence
Pseudoxanthomonas	Presence	Absence	Absence
Pythium	Presence	Absence	Absence
Quadrisphaera	Presence	Absence	Absence
Ramlibacter	Presence	Absence	Absence
Reyranella	Presence	Presence	Absence
Rhizobium	Presence	Absence	Absence
Rhodobium	Absence	Presence	Absence
Rhodococcus	Presence	Absence	Absence
Rhodocytophaga	Presence	Presence	Absence
Rhodoferax	Presence	Absence	Absence
Rhodopila	Presence	Absence	Absence
Rickettsia	Presence	Absence	Absence
Roseomonas	Presence	Presence	Absence
Rothia	Absence	Presence	Absence
Rubellimicrobium	Presence	Presence	Absence
Rubricoccus	Presence	Absence	Absence
Rubritalea	Presence	Absence	Absence
Rubrivivax	Presence	Presence	Absence
Rugosimonospora	Presence	Absence	Absence
Saccharopolyspora	Presence	Absence	Absence
Saccharothrix	Presence	Presence	Absence
Salinarimonas	Presence	Presence	Absence
Salinibacterium	Presence	Absence	Absence
Sciscionella	Presence	Absence	Absence
Serratia	Absence	Presence	Absence

Continued on next page

Supplementary Table 1 – continued from previous page

Genus	Arid	Margin	Hyperarid
Shewanella	Absence	Presence	Absence
Shigella	Presence	Absence	Absence
Shimazuella	Presence	Absence	Absence
Singulisphaera	Presence	Absence	Absence
Sinomonas	Absence	Presence	Absence
Sinorhizobium	Presence	Presence	Absence
Skermanella	Presence	Presence	Absence
Solimonas	Presence	Absence	Absence
Solwaraspora	Presence	Presence	Absence
Sorangium	Presence	Absence	Absence
Sphaerisporangium	Presence	Presence	Absence
Sphingopyxis	Presence	Absence	Absence
Sphingosinicella	Presence	Absence	Absence
Spirillospora	Presence	Absence	Absence
Sporichthya	Presence	Presence	Absence
Sporocytophaga	Presence	Presence	Absence
Sporolactobacillus	Presence	Absence	Absence
Sporosarcina	Presence	Absence	Absence
Steroidobacter	Presence	Presence	Absence
Streptosporangium	Presence	Absence	Absence
Sulfitobacter	Presence	Presence	Absence
Tatlockia	Presence	Absence	Absence
Terracoccus	Presence	Absence	Absence
Thermacetogenium	Absence	Presence	Absence
Thermobispora	Presence	Absence	Absence
Thermocrispum	Presence	Absence	Absence
Thermomonas	Presence	Absence	Absence
Thermus	Presence	Presence	Absence
Thioalkalivibrio	Presence	Absence	Absence
Trachelomonas	Presence	Absence	Absence
Turicibacter	Presence	Absence	Absence
Uliginosibacterium	Presence	Absence	Absence
Umezawaea	Presence	Absence	Absence
Variovorax	Presence	Presence	Absence
Verrucomicrobium	Presence	Absence	Absence
Virgisporangium	Presence	Presence	Absence
Woodsholea	Presence	Absence	Absence
Xylanimicrobium	Absence	Presence	Absence
Yonghaparkia	Presence	Absence	Absence

References

[1] Yi-Juan Hu and Glen A Satten. Testing hypotheses about the microbiome using the linear decomposition model (ldm). *Bioinformatics*, 36(14):4106–4115, 2020.

[2] Emily S Charlson, Jun Chen, Rebecca Custers-Allen, Kyle Bittinger, Hongzhe Li, Rohini Sinha, Jennifer Hwang, Frederic D Bushman, and Ronald G Collman. Disordered microbial communities in the upper respiratory tract of cigarette smokers. *PloS one*, 5(12):e15216, 2010.

[3] Sture Holm. A simple sequentially rejective multiple test procedure. *Scandinavian journal of statistics*, pages 65–70, 1979.

[4] Yoav Benjamini and Yosef Hochberg. Controlling the false discovery rate: a practical and powerful approach to multiple testing. *Journal of the Royal statistical society: series B (Methodological)*, 57(1):289–300, 1995.

[5] Changwon Lim, Pranab K Sen, and Shyamal D Peddada. Robust analysis of high throughput screening (hts) assay data. *Technometrics*, 55(2):150–160, 2013.

[6] Leo Lahti, Jarkko Salojärvi, Anne Salonen, Marten Scheffer, and Willem M De Vos. Tipping elements in the human intestinal ecosystem. *Nature communications*, 5(1):4344, 2014.
