# Supplementary material for: Multigroup analysis of compositions of microbiomes with covariate adjustments and repeated measures
Source: Nat Methods. 2023 Dec 29;21(1):83–91. doi: 10.1038/s41592-023-02092-7 (PMC10776411; doi:10.1038/s41592-023-02092-7)
Supplement: Supplementary file 2 — Reporting Summary [file 41592_2023_2092_MOESM2_ESM.pdf]

nature portfolio

Corresponding author(s): Shyamal Peddada

Last updated by author(s): Sep 13, 2023

Reporting Summary

Nature Portfolio wishes to improve the reproducibility of the work that we publish. This form provides structure for consistency and transparency in reporting. For further information on Nature Portfolio policies, see our Editorial Policies and the Editorial Policy Checklist.

Statistics

For all statistical analyses, confirm that the following items are present in the figure legend, table legend, main text, or Methods section.

n/a	Confirmed	
☐	☒	The exact sample size (*n*) for each experimental group/condition, given as a discrete number and unit of measurement
☐	☒	A statement on whether measurements were taken from distinct samples or whether the same sample was measured repeatedly
☐	☒	The statistical test(s) used AND whether they are one- or two-sided<br>*Only common tests should be described solely by name; describe more complex techniques in the Methods section.*
☐	☒	A description of all covariates tested
☐	☒	A description of any assumptions or corrections, such as tests of normality and adjustment for multiple comparisons
☐	☒	A full description of the statistical parameters including central tendency (e.g. means) or other basic estimates (e.g. regression coefficient) AND variation (e.g. standard deviation) or associated estimates of uncertainty (e.g. confidence intervals)
☐	☒	For null hypothesis testing, the test statistic (e.g. *F*, *t*, *r*) with confidence intervals, effect sizes, degrees of freedom and *P* value noted<br>*Give P values as exact values whenever suitable.*
☒	☐	For Bayesian analysis, information on the choice of priors and Markov chain Monte Carlo settings
☒	☐	For hierarchical and complex designs, identification of the appropriate level for tests and full reporting of outcomes
☐	☒	Estimates of effect sizes (e.g. Cohen's *d*, Pearson's *r*), indicating how they were calculated

Our web collection on statistics for biologists contains articles on many of the points above.

Software and code

Policy information about availability of computer code

Data collection	No software was used for data collection.
Data analysis	ANCOM-BC2 has been implemented in the R package ANCOMBC, which is available on Bioconductor at https://www.bioconductor.org/packages/release/bioc/html/ANCOMBC.html. The entirety of the analyses, conducted in RStudio (R version 4.2.2) on aarch64-apple-darwin20 (64-bit) running macOS Monterey 12.6.8, with the sole exception of the soil microbiome richness trend test, is provided in the related GitHub repository, which can be found at https://github.com/FrederickHuangLin/ANCOM-BC2-Code-Archive. For the aforementioned exception, ORIOGEN 4.01 was employed and is available at https://www.niehs.nih.gov/research/resources/software/biostatistics/oriogen/index.cfm.

For manuscripts utilizing custom algorithms or software that are central to the research but not yet described in published literature, software must be made available to editors and reviewers. We strongly encourage code deposition in a community repository (e.g. GitHub). See the Nature Portfolio guidelines for submitting code & software for further information.

Data

Policy information about availability of data

All manuscripts must include a data availability statement. This statement should provide the following information, where applicable:

- Accession codes, unique identifiers, or web links for publicly available datasets
- A description of any restrictions on data availability
- For clinical datasets or third party data, please ensure that the statement adheres to our policy

The upper respiratory tract (URT) data were sourced from the LOCOM R package (https://github.com/yijuanhu/LOCOM-Archive). The Quantitative Microbiome Project (QMP) data are accessible via the SPRING R package (https://github.com/GraceYoon/SPRING) or the ANCOMBC package (https://www.bioconductor.org/packages/release/bioc/html/ANCOMBC.html). Data pertaining to soil microbiome for aridity and gut microbiome in IBD patients are hosted on Qiita, with respective links available at https://qiita.ucsd.edu/study/description/10360 and https://qiita.ucsd.edu/study/description/11546, respectively.

Human research participants

Policy information about studies involving human research participants and Sex and Gender in Research.

Reporting on sex and gender	No experiment in this study
Population characteristics	No experiment in this study
Recruitment	No experiment in this study
Ethics oversight	No experiment in this study

Note that full information on the approval of the study protocol must also be provided in the manuscript.

Field-specific reporting

Please select the one below that is the best fit for your research. If you are not sure, read the appropriate sections before making your selection.

☒ Life sciences ☐ Behavioural & social sciences ☐ Ecological, evolutionary & environmental sciences

For a reference copy of the document with all sections, see nature.com/documents/nr-reporting-summary-flat.pdf

Life sciences study design

All studies must disclose on these points even when the disclosure is negative.

Sample size	We utilized two publicly available datasets: soil microbiome for aridity and gut microbiome in IBD patients. No sample size calculation was performed.
Data exclusions	We utilized all samples provided by the authors on the public website for the two datasets. No exclusions were made.
Replication	Not relevant. We used publicly available datasets and did not participate in sample generation.
Randomization	Not relevant. For this paper, we utilized two publicly available datasets: soil microbiome for aridity and gut microbiome in IBD patients. We did not conduct any randomization.
Blinding	Not relevant. Two real datasets used for illustrations in this paper, i.e., soil microbiome for aridity and gut microbiome in IBD patients, are publicly available. We were not involved in the blinding or unblinding process.

Reporting for specific materials, systems and methods

We require information from authors about some types of materials, experimental systems and methods used in many studies. Here, indicate whether each material, system or method listed is relevant to your study. If you are not sure if a list item applies to your research, read the appropriate section before selecting a response.

Materials & experimental systems

n/a	Involved in the study
☒	☐ Antibodies
☒	☐ Eukaryotic cell lines
☒	☐ Palaeontology and archaeology
☒	☐ Animals and other organisms
☒	☐ Clinical data
☒	☐ Dual use research of concern

Methods

n/a	Involved in the study
☒	☐ ChIP-seq
☒	☐ Flow cytometry
☒	☐ MRI-based neuroimaging
